# Scanner calibration revisited

**DOI:** 10.1186/1471-2105-11-361

**Published:** 2010-07-01

**Authors:** Alexander E Pozhitkov

**Affiliations:** 1 Max Plank Institute for Evolutionary Biology. August-Thienemann-Str-2, 24306, Ploen, Germany

## Abstract

**Background:**

Calibration of a microarray scanner is critical for accurate interpretation of microarray results. Shi et al. (*BMC Bioinformatics*, 2005, **6**, Art. No. S11 Suppl. 2.) reported usage of a Full Moon BioSystems slide for calibration. Inspired by the Shi et al. work, we have calibrated microarray scanners in our previous research. We were puzzled however, that most of the signal intensities from a biological sample fell below the sensitivity threshold level determined by the calibration slide. This conundrum led us to re-investigate the quality of calibration provided by the Full Moon BioSystems slide as well as the accuracy of the analysis performed by Shi et al.

**Methods:**

Signal intensities were recorded on three different microarray scanners at various photomultiplier gain levels using the same calibration slide from Full Moon BioSystems. Data analysis was conducted on raw signal intensities without normalization or transformation of any kind. Weighted least-squares method was used to fit the data.

**Results:**

We found that initial analysis performed by Shi et al. did not take into account autofluorescence of the Full Moon BioSystems slide, which led to a grossly distorted microarray scanner response. Our analysis revealed that a power-law function, which is explicitly accounting for the slide autofluorescence, perfectly described a relationship between signal intensities and fluorophore quantities.

**Conclusions:**

Microarray scanners respond in a much less distorted fashion than was reported by Shi et al. Full Moon BioSystems calibration slides are inadequate for performing calibration. We recommend against using these slides.

## Introduction

Shi et al. [1] published a paper in BMC Bioinformatics about the need to calibrate microarray scanners. Specifically, they reported a bias in microarray gene expression data if the scanner used to interpret signal intensity was not properly calibrated using a Full Moon BioSystems slide (Sunnyvale, CA, USA). This calibration slide is made of glass spotted with Cy3 and Cy5 dyes at various concentrations. Using this slide, a scanner calibration procedure does not involve any target hybridization. In our subsequent research, we followed their recommendations, converting signal intensities to actual number of fluorophores [2]. Upon calibrating a new Agilent scanner, however, we noticed that most of the probe signal intensities were well below the sensitivity threshold determined by the calibration slide. A representative of Full Moon BioSystems revealed that the "buffer", used to make spots on their calibration slide, autofluoresces. This new understanding lead us to further investigate the utility of calibration slides (discussed below).

Our experiments show the sensitivity and linearity of the photomultipler tube (PMT) is significantly underestimated by the Full Moon BioSystems calibration slide. Because understanding the scanner response is crucial for the meaningful interpretation of microarrays, we feel it is appropriate to communicate this note to the readers, especially in light of the fact that the Shi et al. paper has been well cited.

## Background

Based on the text of Shi et al. [1] paper, we assume that they were not aware of the autofluorescence problem. Nor does it appear that they were aware the fact that a discrepancy exists in the assigned concentrations of the dye within the spots in the Full Moon BioSystems documentation. Full Moon BioSystems provides a "User's manual" and a layout file. The manual schematically shows spots, which form a rectangular pattern of rows and columns. The columns contain replicated spots and the manual assigns dye concentration to each column (Table 1). The layout file, so called GAL file, provides coordinates for each spot and indicates which dye it is. The layout file is usually used by the scanner software. Here, we investigated the content of this file in a text editor. While the manual provides a description table with column 28 referring to the "buffer" spots (see Table 1, note asterisk marking), the layout file for the same spots indicates a certain concentration of dye (note asterisk marking, Table 2). It is our understanding that the "buffer" spots were supposed to be negative controls (*i.e*. a buffer spotted without the dye).

**Table 1:** Calibration slide description provided by Full Moon BioSystems.

Series (Column No.)	Fluorophores/um^2^	Series (Column No.)	Fluorophores/um^2^
1	1.47E + 05	17	2.24E+00
2	7.35E + 04	18	1.12E+00
3	3.68E + 04	19	5.61E-01
4	1.84E + 04	20	2.80E-01
5	9.19E + 03	21	1.40E-01
6	4.59E + 03	22	7.01E-02
7	2.30E + 03	23	3.50E-02
8	1.15E + 03	24	1.75E-02
9	5.74E + 02	25	8.76E-03
10	2.87E + 02	26	4.38E-03
11	1.44E + 02	27	2.19E-03
12	7.18E + 01	*28*	*Buffer*
13	3.59E + 01	29	0
14	1.79E + 01	30	0
15	8.97E + 00	31	0
16	4.49E + 00	32	Position Marker

**Table 2:** Layout file downloaded from the Full Moon BioSystems web site.

Column	Name	ID
1	Marker	Cy3
2	0	Cy3
3	0	Cy3
4	0	Cy3
*5*	*1.10E-03*	Cy3
6	2.19E-03	Cy3
7	4.38E-03	Cy3
8	8.76E-03	Cy3
...	...	...

## Methods

Full Moon BioSystems calibration slide was scanned by three different scanners: Agilent, Bio-Rad and GenePix. Prior scanning, no hybridization was conducted, because the calibration slide already carries spots of dyes. With Agilent and Bio-Rad both color channels were investigated. In addition, several PMT settings were tested on the Agilent scanner.

## Revised Calibration Model

**Figure 1:**
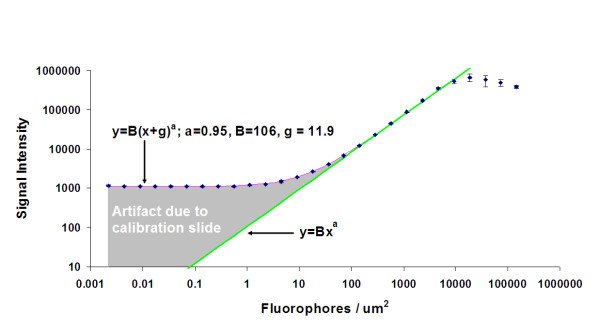
**Photomultiplier response of the Agilent scanner, Cy3 channel**. Photomultiplier gain is 100.

Given that signal intensity vs. concentration curves partially linearize in log-log coordinates (not shown), we propose the following model of scanner response:(1)

where *SI *is signal intensity, *x *- surface density, *g *- autofluorescence of the buffer, *B *and *a *- empirical parameters. This model was used to fit the newly obtained data by an Agilent scanner as well as previously recorded data by Bio-Rad and GenePix scanners. An example of fitting is shown in Figure 1 (Agilent scanner, green channel, PMT = 100).

A magenta line shows the model prediction, while the green line theoretically represents scanner response without the "buffer" interference. It is important to note that because of the extremely large span of data in both *x *and *y *dimensions, a regular least-squares fit is inadequate. A modified merit function was found to be the best for the given data:(2)

The model fitting was conducted by varying parameters until a minimum of *E *was attained. Table 3 shows the fitted parameters for the above mentioned scanners.

**Table 3:** Characteristics of microarray scanners.

Scanner	Channel	PMT^i^	*B*	*a*	g^ii^
Agilent	Cy3	100	106	0.95	11.90
Agilent	Cy3	50	52	0.95	12.14
Agilent	Cy3	20	21	0.94	12.12
Agilent	Cy5	100	54	0.98	3.90
Agilent	Cy5	50	28	0.98	4.04
Agilent	Cy5	20	11	0.99	4.76
GenePix	Cy3	500	208	0.74	7.45
Bio-Rad	Cy3	500	4	0.84	8.33
Bio-Rad	Cy5	500	11	0.86	3.85

^i ^PMT settings are particular for each instrument and should not be compared between the instruments.

^ii ^The dimension of the g value is in Fluorophores/um^2^.

In non-transformed coordinates, the new model introduces two parameters: *B*, the sensitivity of the photomultiplier and *a*, the curvature of the line. It is important not to confuse the slope in our model (*B*) with the slope reported by Shi et al., because the latter is in log-log coordinates, which corresponds to our parameter *a*. Although Figure 1 shows log-scaled coordinates for convenience of viewing, the log transformation was not used to treat the data. Also, the curvature parameter *a *means deviation from the straight line (*a *= 1). As our model predicts, the lower plateau does not apply to a PMT in reality (see Figure 1). The plateau is an artifact of the calibration slide. Hence there is no gross nonlinearity of the PMT response at the lower range of intensities. What distinguishes our study from the work of Shi et al. is that we provided a model that discovered two important properties of scanners: sensitivity (*B*) and curvature (*a*).

The reason why the above mentioned finding is relevant to the discussion of the Shi et al. paper is that calibration of a microarray scanner with Full Moon BioSystems slide is confounding the situation. The calibration slide introduces an artifact of the lower plateau. Moreover, one can see from the Shi et al. study that the span of the linear part (in log-log coordinates) of the PMT response curve depends on the PMT gain. Specifically, at the high gain, the linear part is short, same as at the low PMT gain. We argue that it is because of the lower plateau artifact the linear part is affected. At low PMT gain, the signal is drowned in background fluorescence, while at high gain, the background fluorescence is saturating the PMT. It may very well be that the linear (or slightly curvy) response line spans over much larger range of concentrations than it is reported by Shi et al. This explains why most of the signals were below the lower plateau in our ongoing gene expression studies. In retrospect, the only reason for calibrating a scanner could be to determine the saturation limit of the PMT.

**Figure 2:**
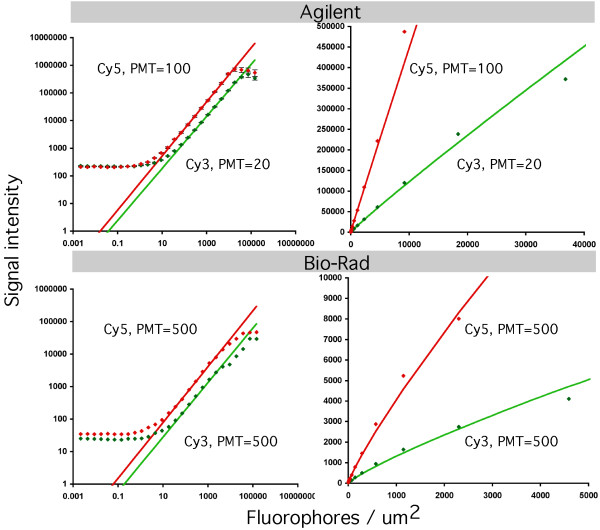
**Comparison of PMT responses for different channels and gains for Agilent (top row) and Bio-Rad (bottom row) scanners**. Left column shows data in log-log coordinates, right column - in original non-transformed coordinates.

The secondary finding of this study is that the curvature of the calibration line (parameter *a*) does not significantly depend on the PMT gain and channel. For the scanners investigated (Agilent, Bio-Rad), the curvature of the line does not differ markedly between Cy3 and Cy5 channels (Table 3). The gain of the photomultiplier (i.e., voltage on the PMT tube) also does not influence the curvature of the lines for the Agilent scanner. Shi et al. claims that there are "inherent differences" in the Cy3 and Cy5 channels for the same PMT setting, which are attributed to "slope". It is important to emphasize once again that the "slope" in terms of Shi et al. is different from the slope in our model. In Shi et al., the slope refers to the linear part of the line in log-log coordinates. In this paper, the slope refers to the parameter *B*, which is a slope in non-transformed coordinates. Presumably, they were also referring to the lower plateaus with regard of the "inherent differences". We agree with them that the PMTs are differing in sensitivities (parameter *B*), but the curvatures of the lines (parameter *a*) are essentially the same. Given that fact, simple ratios of signal intensities between red and green channels for Agilent and Bio-Rad scanners will be perfectly sufficient to describe gene expression changes without calibration. Figure 2 shows comparison between PMT settings and channels for Agilent and Bio-Rad scanners. On the left, signals are plotted in log-log coordinates, on the right - in non-transformed coordinates. The top row of the Figure 2 compares two different channels and two different PMT settings, which is similar to Shi et al, Figure 2D.

Table 3 provides values for the autofluorescence (parameter *g*). Our model accurately captured the value of autofluorescence, because, as one would expect, the parameter *g *is independent on the PMT setting. The *g *values for other scanners are somewhat different from that of Agilent, which is because the calibration slides were from different batches. The *g *value itself (~8-12) is about 4 orders of magnitude larger than the lowest concentration of the dye on the slide. Obviously, with this level of autofluorescence the dilution series 15-27 have no utility (see Table 1).

There seems to be a particular PMT behavior of the Bio-Rad scanner, *i.e*. Figure 2, bottom left, shows a shoulder on the Cy3 curve. To the contrary, GenePix scanner does not display such a behavior. Specifically, the GenePix Cy3 curve is similar to that of Agilent (data not shown).

We propose that a new type of calibration slide with zero autofluorescence be developed and a model be devised that exactly describes behavior of the PMT.

**Note: **our analysis was based on purely raw signal intensities. In other words, none of the data values were normalized, removed or transformed.

## Conclusions

Our study revealed two important facts. First, the lower plateau is an artifact introduced by the Full Moon BioSystems calibration slide. Second, the PMT response curvature is not significantly different between color channels and the curvature is the same for different PMT gains (voltages). We recommend against using Full Moon BioSystems calibration slides.

## Authors' contributions

Alexander Pozhitkov conducted the research and wrote the manuscript.
